# Distinct Genetic Lineages of *Bactrocera caudata* (Insecta: Tephritidae) Revealed by COI and 16S DNA Sequences

**DOI:** 10.1371/journal.pone.0037276

**Published:** 2012-05-17

**Authors:** Phaik-Eem Lim, Ji Tan, I. Wayan Suana, Praphathip Eamsobhana, Hoi Sen Yong

**Affiliations:** 1 Institute of Biological Sciences, University of Malaya, Kuala Lumpur, Malaysia; 2 Institute of Ocean and Earth Sciences, University of Malaya, Kuala Lumpur, Malaysia; 3 Faculty of Science and Mathematics, Mataram University, Mataram, Indonesia; 4 Department of Parasitology, Faculty of Medicine Siriraj Hospital, Mahidol University, Bangkok, Thailand; American Museum of Natural History, United States of America

## Abstract

The fruit fly *Bactrocera caudata* is a pest species of economic importance in Asia. Its larvae feed on the flowers of Cucurbitaceae such as *Cucurbita moschata*. To-date it is distinguished from related species based on morphological characters. Specimens of *B. caudata* from Peninsular Malaysia and Indonesia (Bali and Lombok) were analysed using the partial DNA sequences of cytochrome *c* oxidase subunit I (COI) and 16S rRNA genes. Both gene sequences revealed that *B. caudata* from Peninsular Malaysia was distinctly different from *B. caudata* of Bali and Lombok, without common haplotype between them. Phylogenetic analysis revealed two distinct clades, indicating distinct genetic lineage. The uncorrected ‘p’ distance for COI sequences between *B. caudata* of Malaysia-Thailand-China and *B. caudata* of Bali-Lombok was 5.65%, for 16S sequences from 2.76 to 2.99%, and for combined COI and 16S sequences 4.45 to 4.46%. The ‘p’ values are distinctly different from intraspecific ‘p’ distance (0–0.23%). Both the *B. caudata* lineages are distinctly separated from related species in the subgenus *Zeugodacus* – *B. ascita*, *B. scutellata*, *B. ishigakiensis*, *B. diaphora*, *B. tau*, *B. cucurbitae*, and *B. depressa*. Molecular phylogenetic analysis indicates that the *B. caudata* lineages are closely related to *B. ascita* sp. B, and form a clade with *B. scutellata*, *B. ishigakiensis*, *B. diaphora* and *B. ascita* sp. A. This study provides additional baseline for the phylogenetic relationships of *Bactrocera* fruit flies of the subgenus *Zeugodacus*. Both the COI and 16S genes could be useful markers for the molecular differentiation and phylogenetic analysis of tephritid fruit flies.

## Introduction

Fruit flies of the family Tephritidae are represented by over 4400 species worldwide [1]. Some 200 species are considered pests, causing direct losses to a wide variety of fruit, vegetable and flower crops [2]. The larvae of about 35% of the species attack soft fruits, and about 40% of species develop in the flowers of Asteraceae [3].

In the Oriental Region, fruit flies of the genus *Bactrocera* – previously referred to the genus *Dacus* [4], [5] – are of great economic and agriculture importance because of damage caused to commercial fruits and vegetables. Some 22 species have been listed as of economic importance in Asia [6]. Among these species, *Bactrocera caudata* (Fabricius) had not been recorded in the Australasian and Oceanian regions [5].

*Bactrocera caudata* has a Paleartic and Oriental distribution. It occurs in India, Sri Lanka, Myanmar, Thailand, Vietnam, China, Malaysia, Brunei and Indonesia (Sumatra, Java, Flores) [2]. It is a member of the subgenus *Zeugodacus* and a pest of commercial and edible flowers. *Zeugodacus* is almost exclusively associated with the flowers and fruits of Cucurbitaceae [3]. Specimens of *B. caudata* had been reared from flowers of pumpkin *Cucurbita moschata* in Peninsular Malaysia [7]. To-date there are no additional reports on the host plants of this fruit fly.

Known also as *Dacus caudatus* Fabricius and *Bactrocera maculipennis* Doleschall, *B. caudata* is recognized from other *Zeugodacus* with three postsutural yellow vittae by possession of a transverse black band across the furrow of the face, two pairs of scutellar bristles, and the costal band slightly enlarged at the apex [2], [7]. The males are attracted to cue-lure.

Compared to other members of the *Zeugodacus* group, little attention has been given to the study on the genetic variation in *B. caudata*. In a study of Peninsular Malaysian *B. caudata* involving 14 gene-enzyme systems with 17 loci, the proportion of polymorphic loci was P = 0.41 and the mean heterozygosity was H = 0.11 [8]. To-date the molecular and phylogenetic studies involving *B. caudata* used only a single individual and from a single locality, e.g. Ranong, Thailand [9], Brunei [10], and Chongqing region, China [11]. Genetic information on *B. caudata* from various geographical areas of its distribution range also appear to be lacking.

The present study examined the DNA sequences of COI and 16S rRNA genes in several populations of *B. caudata* from Peninsular Malaysia and Indonesia (Bali and Lombok). These two mitochondrial genes have been commonly used for the study of the phylogenetics of *Bactrocera* species [9]–[15]. Furthermore, mitochondrion DNA markers possess simple structure, uniform organization of the genome, lack of recombination, and with maternal inheritance and relatively rapid evolutionay rates [13], [16], [17]. The resulting COI and 16S sequences revealed the occurrence of distinct genetic lineages in this fruit fly. They are genetically distinct from closely related species of the subgenus *Zeugodacus*.

## Materials and Methods

### Ethics Statement

No specific permits were required for the described field studies. The *Bactrocera* fruit flies are collected in gardens and not from any national parks or protected areas. No specific permissions were required as the locations were in abandoned areas or in campus gardens. The *Bactrocera* species are agricultural pests and are not endangered or protected species.

### Specimens

Adult male *B. caudata* were collected by means of the sex attractant cue-lure (4-[4-(acetyloxy) phenyl]-2-butanone) obtained from Sigma. A small amount of this lure was applied on the upper surface of a green leaf. Fruit flies attracted to the lure were collected with the aid of specimen tubes and plastic bags. The lure remained effective for many hours. A related species, *B. tau* was hatched from infested fruits of *Momordica charantia* (bitter gourd) collected at University of Malaya campus. As outgroups, *Dacus* (*Callantra*) *longicornis* (Dlon1) was collected by cue-lure in Perlis, and *Dacus* sp. (Dlon2) from Gombak, Peninsular Malaysia. The specimens were preserved in ethanol and stored in the freezer until use. Identification of the fruit flies was based on available literature [2], [3], [7] and personal experience (H.S. Yong).

Specimens of *B. caudata* were collected from Peninsular Malaysia: University of Malaya campus, Kuala Lumpur (Bcau1, Bcau2, Bcau18); Clearwater Sanctuary, Perak (Bcau3-Bcau5, Bcau8, Bcau10); Gombak, Selangor (Bcau16); Carey Island, Selangor (Bcau11, Bcau14); Mentakab, Pahang (Bcau9); Dungun, Terengganu (Bcau7); Penang Hill and Georgetown, Penang (Bcau19-Bcau21); and Indonesia: Bali (Bcau15); Gili Meno and Sekotong, Lombok (Bcau12, Bcau13, Bcau17).

### DNA Extraction, Polymerase Chain Reaction, and Sequencing

The genomic DNAs were isolated from two legs or thorax of each adult fruit fly preserved in absolute ethanol using i-genomic CTB DNA Extraction Mini Kit (iNtRON Biotechnology, Inc, Korea).

The partial sequences of COI were amplified and sequenced using the primer combination of UEA7-5′-TACAGTTGGAATAGACGTTGATAC-3′ and UEA10-5′-CCAATGCACTAATCTGCCATATTA-3′ [18]. For 16S rDNA, the primer set of (16S-F) LR-J-13756 5′-TAGTTTTTTTAGAAATAAATTTAATTTA-3′ and (16S-R) LR-N-13308 5′-GCCTTCAATTAAAAGACTAA-3′ [10] was used.

PCR amplification of both molecular markers was carried out using MultiGene Gradient Thermal Cycler (Labnet, USA). The total volume for the PCR amplification was 50 µL consisting of 5.0 µL of 10× *i*-Taq™ *plus* buffer, 5.0 µL of dNTP mixture (2.5 mM each), 0.25 µM of each primer, 1.0 unit of *i*-Taq™ *plus* DNA polymerase, and 50 pg to 1.0 µg DNA. The parameters of PCR amplification were: 3 min at 95°C, followed by 30 cycles of denaturation at 94°C for 1 min, annealing at 50°C for 1 min, extension at 72°C for 1 min, and a final extension at 72°C for 10 min.

PCR products were assayed by electrophoresis on 1.0% agarose mini gel stained with SYBR®Safe DNA gel stain (Invitrogen, USA) and visualized under UV light. The target DNA fragments were isolated and purified by the LaboPass™ PCR purification kit (Cosmo Genetech, South Korea). The purified PCR products were sent to a commercial company for sequencing. Samples were sequenced using BigDye® Terminator v3.1 Sequencing Kit and analyzed on ABI PRISM® 377 Genetic Analyzer. Cycle sequencing conditions were as follows: 25 cycles of 96°C for 10 sec, 50°C for 5 sec and 60°C for 4 min at rapid thermal ramp of 1°C/sec. Samples were purified by Ethanol/EDTA/Sodium Acetate precipitation. The control DNA sequence used was the pGEM-3Zf (+) control template with M13F (−29) control primer.

Representative sequences of this study namely, *B. caudata*: Bcau14, Bcau2 (from Peninsular Malaysia), Bcau12, Bcau15 (from Indonesia); *B. tau*: Btau28; and *D. longicornis*: Dlon1 were deposited in the GenBank. The assigned GenBank accessions numbers are: For COI – Bcau14 (JN542416), Bcau2 (JN542417); Bcau12 (JN542418), Bcau15 (JN542419), Btau28 (JN542420), and Dlon1 (JN542421); for 16S – Bcau14 (JN542422), Bcau2 (JN542423); Bcau12 (JN542424), Bcau15 (JN542425), Btau28 (JN542426), and Dlon1 (JN542427).

### DNA Sequences From Genbank

To compare the genetic diversity of closely related and other *Batrocera* species, both COI and 16S rDNA of mitrochondrial encoded genes were downloaded from the GenBank. The COI sequences obtained from the GenBank were: (1) *Bactrocera caudata* FJ903493; (2) *Bactrocera caudata* GQ458048; (3) *Bactrocera caudata* AF423109; (4) *Bactrocera ascita* sp. A AF423108; (5) *Bactrocera ascita* sp. B AF423111; (6) *Bactrocera scutellata* AY53891; (7) *Bactrocera ishigakiensis* AY530902; (8) *Bactrocera diaphora* GQ458043; (9) *Bactrocera tau* Type A AF400067; (10) *Bactrocera cucurbitae* FJ903497; (11) *Bactrocera cucumis* AY530906; and (12) *Bactrocera depressa* AB192453. The 16S rDNA sequences downloaded from the GenBank were: (1) *Bactrocera caudata* AY037363; (2) *Bactrocera ishigakiensis* AB035099; (3) *Bactrocera scutellata* AB035106; (4) *Bactrocera tau* typeA AB048745; (5) *Batrocera cucurbitae* AY037350; and (6) *Bactrocera cucumis* Type A AB074018.

### Genetic Divergence

To assess the level of variation in the COI and 16S rDNA among the selected samples of different taxa, uncorrected (p) pairwise genetic distances were estimated using PAUP* 4.0b10 software [19].

### Haplotype Network Reconstruction

The genetic diversity or haplotype networks of *B. caudata* were analysed using TCS 1.13 [20] to calculate the minimum number of mutational steps by which the sequences can be joined with >95% confidence. The minimum number of mutational steps required to connect the different groups of haplotypes obtained using the Templeton *et al.* [21] method was identified using the fix connection limit option, as implemented in TCS software. Three separate data sets were carried out for network estimations: (1) all the COI *Bactrocera* sequences obtained from this study and sequences from GenBank; (2) all the 16S rDNA *Bactrocera* sequences obtained from this study and GenBank; and (3) combined sequences of COI and 16S rDNA from this study.

### Sequence Alignment And Phylogenetic Analysis

The COI and 16S rDNA sequences were preliminarily aligned using the CLUSTAL X program [22] and subsequently manually aligned. The sequences of the COI and 16S rDNA were also combined to further understand the systematic relationships among *B. caudata* and closely related species. Several researchers suggested the need to use the incongruence of length differential (ILD) test or partition homogeneity test to determine whether the sequences contain congruent phylogenetic information [23], [24]. In this study, partition homogeneity tests [25]–[27] were performed in PAUP* 4.0b10 software [19] with 100 replicates, heuristic search using the tree-bisection-reconnection (TBR) branch swapping algorithm. Due to some recent criticism against the application of the ILD [28], additional analyses on each gene were conducted for topology comparison.

The aligned sequences were subjected to maximum-parsimony (MP) and neighbour-joining (NJ) analyses using PAUP* 4.0b10 [19]. The MP tree was constructed using the heuristic search option, 100 random sequences additions, tree bisection reconnection (TBR) branch swapping, and unordered and unweighted characters. Bootstrap percentage (BP) was computed with 1000 replications. NJ bootstrap values were estimated using 1000 replicates with Kimura's two-parameter model of substitution (K2P distance) evolution model.

Maximum likelihood (ML) analysis was performed by Treefinder version October 2008 [29]. Bayesian (BI) analysis was performed using MrBayes 3.1.2 [30]. The best fit nucleotide substitution model was determined using KAKUSAN v.3 [31], which also generates input files for ML and BI. Best fit models were evaluated using the corrected Akaike Information Criterion [32], [33] for ML and the Bayesian Information Criterion (BIC) with significance determined by Chi-square analysis.

The best selected model for COI marker was general time-reversible (GTR) model of DNA evolution with a gamma shape parameter (G); the best selected model for 16S rDNA was J1 model with a gamma shape parameter (G); while the best selected model for the combined sequences of COI and 16S rDNA was J2 model with a gamma shape parameter (G).

ML analyses were performed with 1000 bootstrap replicates. Two parallel runs were performed in MrBayes analysis using four chains of Markov chain Monte Carlo (MCMC). One million Markov chain Monte Carlo (MCMC) generations were run, with convergence diagnostics calculated every 1000th generation for monitoring the stabilization of log likelihood scores. Trees in each chain were sampled every 100th generation. A 50% majority rule consensus tree was generated from the sampled trees after discarding the first 20%.

## Results

### Sequences Alignment and Statistics

The aligned partial sequences of COI consisted of 637 characters; 47 sites were variable and 148 sites were phylogenetically informative. MP analysis yielded one single most parsimonious tree of 447 steps, a consistency index of 0.6130 and retention index of 0.7496. The aligned partial sequences of 16S rDNA consisted of 441 sites; 30 sites were variable and 29 sites were phylogenetically informative. MP analysis produced four single most parsimonious trees of 89 steps, a consistency index of 0.8090 and retention index of 0.8712. The combined partial sequences of COI and 16S rDNA consisted of 1078 characters; 124 sites were variable and 101 sites were phylogenetically informative. MP analysis yielded one single most parsimonious tree of 309 steps, a consistency index of 0.8770 and retention index of 0.83199.

The PH test for our datasets showed that COI and 16S rDNA as well as the combined data set of COI and 16S rDNA shared the same phylogenetic information, where P = 0.01. Hence combined data sets were used for the phylogenetic analyses.

### Genetic divergence

The uncorrected ‘p’ distances between different species of *Bactrocera* based on COI, 16S rDNA and combined COI and 16s rDNA sequences are summarized in Tables 1 and 2.

**Table 1:** Percentage of uncorrected “p” distance matrix between *Bactrocera caudata* and related species based on 16S (above diagonal) and COI (below diagonal) DNA sequences.

Taxon	1	2	3	4	5	6	7	8	9	10	11	12	13	14	15	16
1. *B. caudata* (Malaysia)	-	N/A	N/A	N/A	N/A	N/A	N/A	N/A	N/A	N/A	N/A	N/A	N/A	N/A	N/A	N/A
2. *B. caudata* (China)	0.00	-	N/A	N/A	N/A	N/A	N/A	N/A	N/A	N/A	N/A	N/A	N/A	N/A	N/A	N/A
3. *B. caudata* (Thailand)	0.00	0.00	-	N/A	N/A	N/A	N/A	N/A	N/A	N/A	N/A	N/A	N/A	N/A	N/A	N/A
4. Bcau14, *B. caudata* (Carey Island)	0.00	0.00	0.00	-	0.23	2.99	2.99	N/A	N/A	2.76	2.76	N/A	N/A	4.15	4.38	4.86
5. Bcau2, *B. caudata* (Univ. Malaya)	0.00	0.00	0.00	0.00	-	2.76	2.76	N/A	N/A	2.53	2.53	N/A	N/A	3.91	4.15	4.62
6. Bcau12, *B. caudata* (Lombok)	5.65	5.65	5.65	5.65	5.65	-	0.00	N/A	N/A	1.84	1.84	N/A	N/A	4.37	5.06	4.62
7. Bcau15, *B.caudata* (Bali)	5.65	5.65	5.65	5.65	5.65	0.00	-	N/A	N/A	1.84	1.84	N/A	N/A	4.37	5.06	4.62
8. *B. ascita* sp. B (Thailand)	7.22	7.22	7.22	7.22	7.22	6.76	6.76	-	N/A	N/A	N/A	N/A	N/A	N/A	N/A	N/A
9. *B. ascita* sp. A (Thailand)	8.79	8.79	8.79	8.79	8.79	9.89	9.89	9.43	-	N/A	N/A	N/A	N/A	N/A	N/A	N/A
10. *B. scutellata* (Japan)	9.11	9.11	9.11	9.11	9.11	8.63	8.63	8.80	7.69	-	0.00	N/A	N/A	3.90	4.13	4.36
11. *B. ishigakiensis* (Japan)	9.42	9.42	9.42	9.42	9.42	8.95	8.95	9.11	8.01	0.47	-	N/A	N/A	3.90	4.13	4.36
12. *B. diaphora* (China)	8.79	8.79	8.79	8.79	8.79	8.63	8.63	9.42	7.38	0.94	1.41	-	N/A	N/A	N/A	N/A
13. *B. depressa* (Japan)	13.97	13.97	13.97	13.97	13.97	13.97	13.97	12.56	14.29	13.19	13.50	13.81	-	N/A	N/A	N/A
14. Btau28, *B, tau* (Univ. Malaya)	11.30	11.30	11.30	11.30	11.30	12.56	12.56	11.79	11.15	11.77	12.24	11.93	13.03	-	1.15	2.29
15. *B. cucurbitae*	12.24	12.24	12.24	12.24	12.24	12.62	12.62	12.27	12.28	12.04	12.41	12.23	14.87	3.78	-	2.98
16. *B. cucumis*	11.77	11.77	11.77	11.77	11.77	12.24	12.24	11.62	11.46	12.72	13.03	12.87	12.72	7.38	8.72	-

NA, not available.

**Table 2:** Percentage of uncorrected “p” distance matrix between *Bactrocera caudata* samples based on combined COI and 16S rDNA sequences.

Taxon	1	2	3	4	5
1. Bcau2, *B. caudata* (University Malaya)	-				
2. Bcau14, *B. caudata* (Carey Island)	0.09	-			
3. Bcau12, *B. caudata* (Lombok)	4.45	4.46	-		
4. Bcau13, *B. caudata* (Lombok)	4.45	4.46	0.00	-	
5. Bcau15, *B. caudata* (Bali)	4.45	4.46	0.00	0.00	-

### Haplotype Network Reconstruction

**Figure 1:**
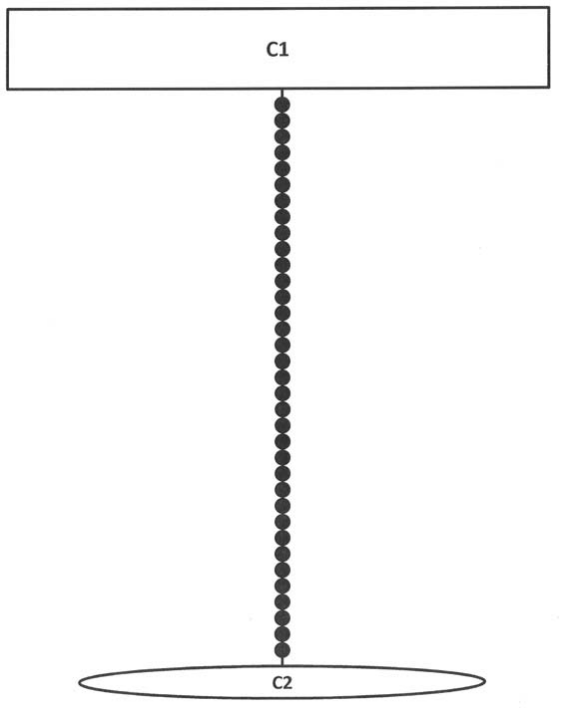
Statistical parsimony networks for COI haplotypes of *Bactrocera caudata*. Lines represent parsimonous connections between haplotypes and the small circles indicate missing haplotype. The size of square or oval corresponds to the haplotype frequency.

The aligned sequences of COI for *B. caudata* consisted of 637 sites. The haplotype network reconstruction showed two divergent groups of haplotypes (Figure 1, Table 3). A minimum of 36 mutational steps was required to link these groups. The haplotype C1 differed from haplotype C2 by 36 changes.

**Table 3:** Variation sites in DNA sequences for mitochondrial COI of *Bactrocera* species from various localities.

Haplotype	Variation sites in DNA sequence																	
	**9**	**84**	**96**	**99**	**102**	**108**	**117**	**120**	**129**	**136**	**153**	**168**	**180**	**216**	**288**	**321**	**325**	**366**
C1	T	T	T	C	T	T	A	C	T	C	A	A	T	C	T	A	T	C
C2	C	A	C	T	C	A	G	A	C	T	T	G	C	A	C	G	C	T
	**372**	**399**	**417**	**438**	**450**	**481**	**483**	**489**	**499**	**546**	**549**	**588**	**594**	**600**	**606**	**615**	**619**	**621**
C1	C	A	T	T	C	T	A	C	T	T	C	C	T	A	C	T	C	A
C2	T	G	C	C	T	C	T	T	C	C	T	T	G	C	T	A	T	G

Haplotype CI consisted of *B. caudata* samples Bcau1, Bcau2, Bcau3, Bcau4, Bcau5, Bcau7, Bcau8, Bcau9, Bcau10, Bacau11, Bcau14, Bcau16, Bcau18, Bcau19, Bcau20 and Bcau21 from Peninsular Malaysia, FJ903493, *B. caudata*, Malaysia, GQ458048, *B. caudata*, China, AF423109, *B. caudata*, Thailand; while C2 consisted of *B. caudata* samples Bcau12, Bcau13, Bcau15 from Indonesia.

**Figure 2:**
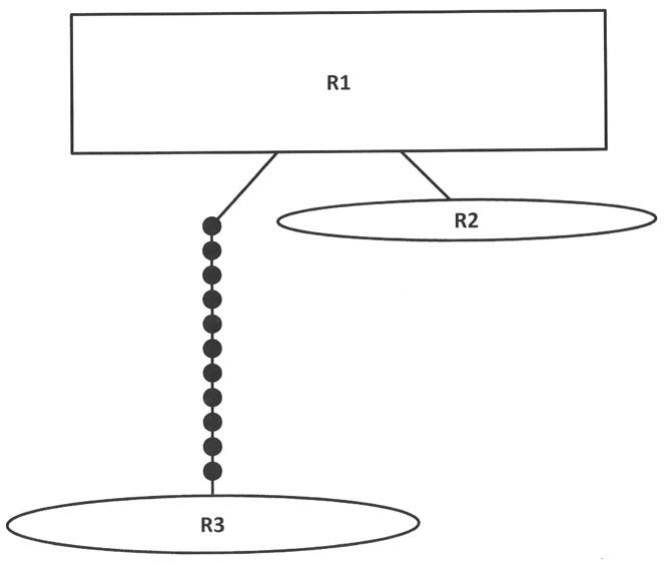
Statistical parsimony networks for 16S rDNA haplotypes of *Bactrocera caudata*. Lines represent parsimonous connections between haplotypes and the small circles indicate missing haplotype. The size of square or oval corresponds to the haplotype frequency.

The aligned sequences of 16S rDNA for *B. caudata* consisted of 435 sites. The haplotype network reconstruction showed three divergent groups of haplotypes (Figure 2, Table 4). A minimum of 12 mutational steps was required to link these groups. The haplotype R1 differed from haplotype R2 by one base pair at site 23 of the aligned sequences and differed from R3 by 12 basepairs. Haplotype R2 differed from R3 by 13 basepairs.

**Table 4:** Variation sites in DNA sequences of *Bactrocera* species for mitochondrial 16S rDNA from various localities.

Haplotype	Variation sites in DNA sequence												
	**23**	**30**	**151**	**160**	**161**	**187**	**206**	**213**	**260**	**325**	**326**	**398**	**420**
R1	C	A	G	C	A	C	A	A	G	C	T	C	T
R2	A	A	G	C	A	C	A	A	G	C	T	C	T
R3	C	G	A	T	C	T	G	G	T	A	C	T	C

Haplotype RI consisted of *B. caudata* samples Bcau1, Bcau2, Bacau3, Bcau4, Bcau5, Bcau7, Bcau8, Bcau9, Bcau10, Bacau11, Bcau16, Bcau18, Bcau19, Bcau20, Bcau21 from Peninsular Malaysia and AY037363, *B. caudata*, Brunei; R2 consisted of Bcau14 from Peninsular Malaysia; while R3 consisted of *B. caudata* samples Bcau12, Bcau13, Bcau15 and Bcau17 from Indonesia.

**Figure 3:**
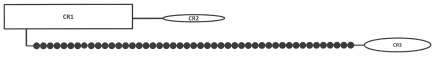
Statistical parsimony networks for combined COI and 16S rDNA haplotypes of *Bactrocera caudata*. Lines represent parsimonous connections between haplotypes and the small circles indicate missing haplotype. The size of square or oval corresponds to the haplotype frequency.

The aligned combined sequences of COI and 16S rDNA for *B. caudata* consisted of 1072 sites. The haplotype network reconstruction showed three divergent groups of haplotypes (Figure 3, Table 5). A minimum of 48 mutational steps was required to link these groups. The haplotype CR1 differed from haplotype CR2 by one change and from haplotype CR3 by 48 changes. Haplotype CR2 differed from CR3 by 49 changes.

**Table 5:** Variation sites in DNA sequences of *Bactrocera* species for mitochondrial COI and 16S rDNA from various localities.

Haplotype	Variation sites in DNA sequence																	
	**9**	**84**	**96**	**99**	**102**	**108**	**117**	**120**	**129**	**136**	**153**	**168**	**180**	**216**	**288**	**321**	**325**	**366**
CR1	T	T	T	C	T	T	A	C	T	C	A	A	T	C	T	A	T	C
CR2	T	T	T	C	T	T	A	C	T	C	A	A	T	C	T	A	T	C
CR3	C	A	C	T	C	A	G	A	C	T	T	G	C	A	C	G	C	T
	**372**	**399**	**417**	**438**	**450**	**481**	**483**	**489**	**499**	**546**	**549**	**588**	**594**	**600**	**606**	**615**	**619**	**621**
CR1	C	A	T	T	C	T	A	C	T	T	C	C	T	A	C	T	C	A
CR2	C	A	T	T	C	T	A	C	T	T	C	C	T	A	C	T	C	A
CR3	T	G	C	C	T	C	T	T	C	C	T	T	G	C	T	A	T	G
	**660**	**667**	**788**	**797**	**798**	**824**	**843**	**850**	**897**	**962**	**963**	**1035**	**1057**					
CR1	C	A	G	C	A	C	A	A	G	C	T	C	T					
CR2	A	A	G	C	A	C	A	A	G	C	T	C	T					
CR3	C	G	A	T	C	T	G	G	T	A	C	T	C					

Haplotype CRI consisted of *B. caudata* samples Bcau1, Bcau2, Bacau3, Bcau4, Bcau5, Bcau7, Bcau8, Bcau9, Bcau10, Bacau11, Bcau16, Bcau18, Bcau19, Bcau20 and Bcau21 from Peninsular Malaysia; CR2 consisted of Bcau14 from Peninsular Malaysia; while CR3 consisted of *B. caudata* samples Bcau12, Bcau13 and Bcau15 from Indonesia.

### Phylogenetic Relationships

All phylogenetic analyses produced the same topology of the phylogenetic trees. They differed only in associations at poorly supported nodes. Only ML trees were presented for the three sets of sequences.

### *Cytochrome* c *oxidase subunit* I (COI)

**Figure 4:**
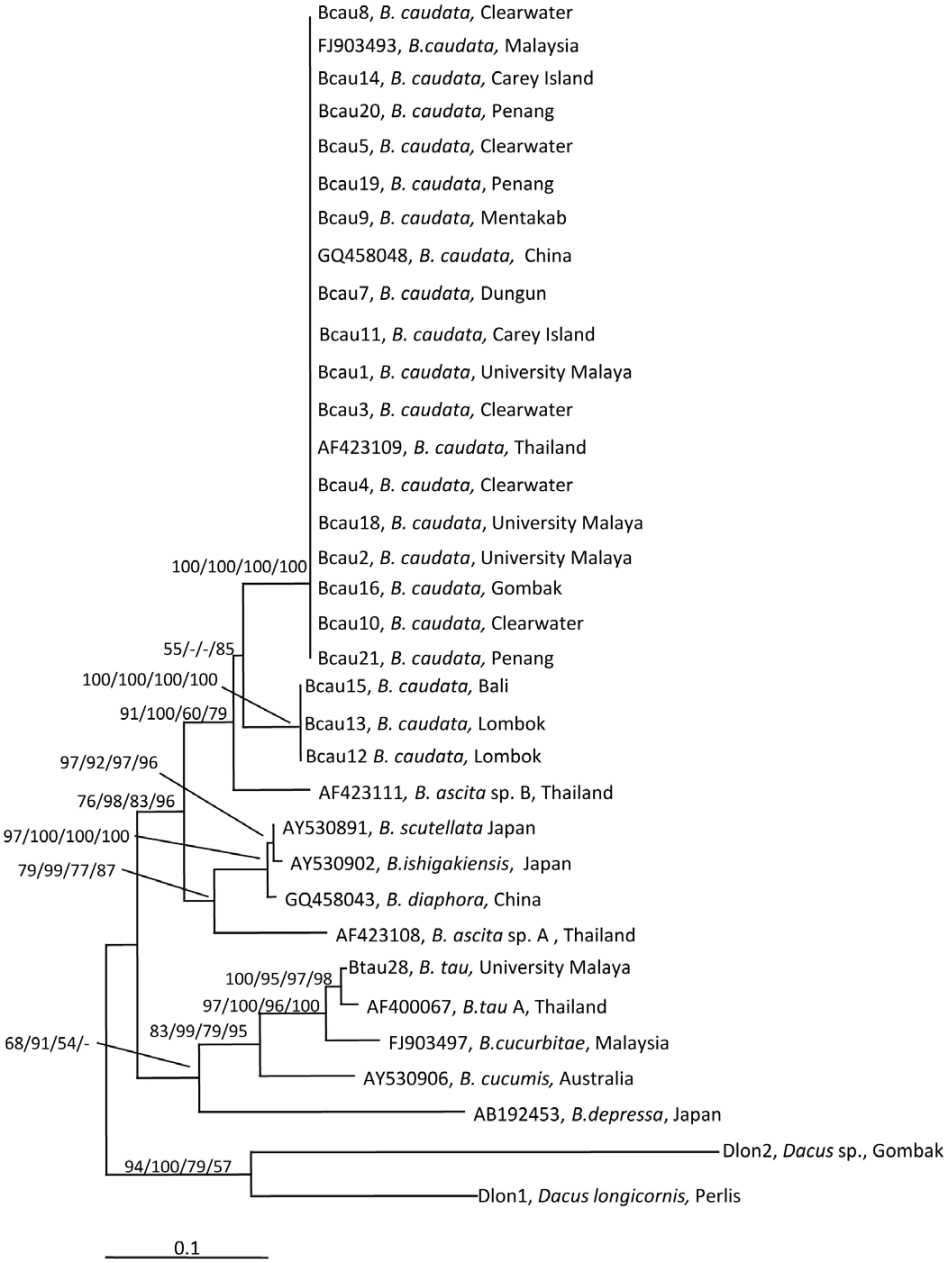
The 50% majority-rule consensus tree resulting from maximum likelihood analysis of partial COI sequences (substitution rate parameters: TC = 0.6520485; TA = 0.1024025; TG = 0.005185725; CA = 0.02170842; CG = 0.001222865; AG = 0.217396). -Ln likelihood was 2742.654. The bootstrap values (ML/Bayesian Inference/MP/NJ) are shown at the branches. Bar indicates substitutions per site.

The COI ML tree consisted of two main groups (Figure 4). The first group, supported with no bootstrap to high bootstrap values (ML = 68%; BI = 91%; MP = 54%), consisted of the following taxa: *B. tau, B. cucurbitae, B. cucumis*, and *B. depressa*.

The second group could be divided into two main subgroups. The first subgroup consisted of *B. scutellata, B. ishigakiensis, B. diaphora* and *B. ascita* sp. A as the most basal species and they were supported with moderate to high bootstrap values of 79% for ML; 99% for BI; 77% for MP and 87% for NJ. The second subgroup consisted of *B. caudata* and *B. ascita* sp. B and supported with low to high bootstrap values (ML = 91%; BI = 100%; MP = 60%; NJ = 79%).The second subgroup was further divided into two main clades: (1) Clade 1 comprising of only *B. ascita* sp. B with no bootstrap support; and (2) Clade 2 comprising *B. caudata* with low to moderate bootstrap support values of 55% for ML and 85% for NJ. Clade 2 was sub-divided into two sub-clades: *B. caudata* from Malaysia, China and Thailand; and *B. caudata* from Bali and Lombok – Nusa Tenggara, Indonesia and were supported with full bootstrap supports for all analyses.

### 16S rDNA

**Figure 5:**
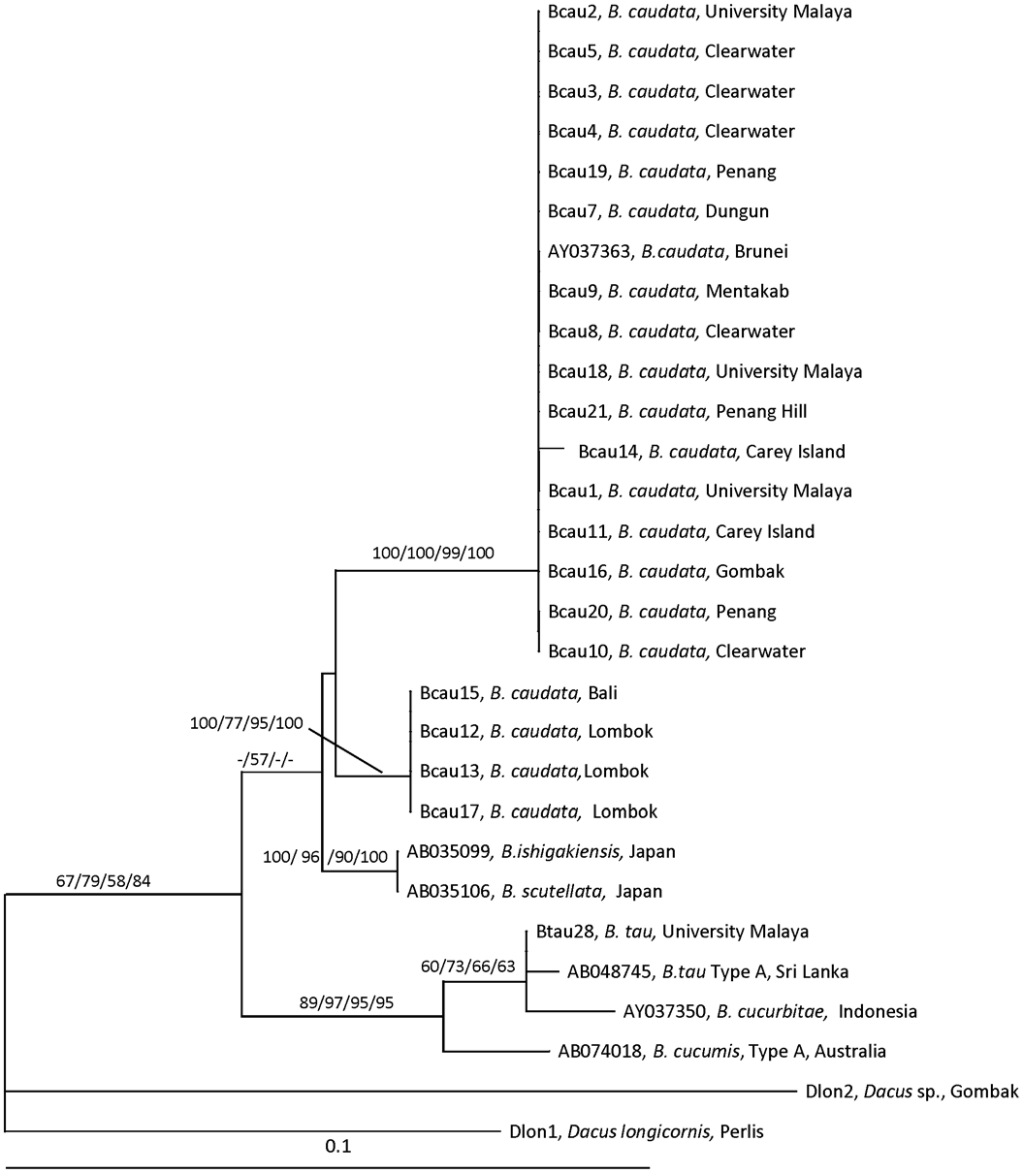
The 50% majority-rule consensus tree resulting from maximum likelihood analysis of partial 16S rDNA sequences (substitution rate parameters: TC = 0.6192997; TA = 0.02629145; TG = 0.02629145; CA = 0.07552851; CG =  = 0.07552851; AG = 0.1770604). -Ln likelihood was 981.8218. The bootstrap values (ML/Bayesian Inference/MP/NJ) are shown at the branches. Bar indicates substitutions per site.

The 16S rDNA ML tree consisted of two main groups supported with low to moderate bootstrap support values (ML = 67%; BI = 79%; MP = 58%; NJ = 84%) (Figure 5). The basal group comprised *B. tau, B. cucurbitae* and *B. cucumis* supported with moderate to high bootstrap values (ML = 89%; BI = 97%; MP = 95%; NJ = 95%). The other group consisted of *B. caudata, B. scutellata*, and *B. ishigakiensis*, with bootstrap value of 57% for BI only. This group is further divided into two main clades: (1) *B. ishigakiensis* and *B. scutellata*; and (2) *B. caudata*. Clade 2 consisted of two sub-clades: sub-clade 1 consisting of *B. caudata* from various localities from Malaysia and one from Brunei supported with high to full bootstrap values (ML = 100%; BI = 100%; MP = 99%; NJ = 100%); and sub-clade 2 consisting of *B. caudata* from Bali and Lombok (Nusa Tenggara, Indonesia) with bootstrap values ML = 100%, BI = 77%, MP = 95%, and NJ = 100%.

### Combined COI and 16S rDNA

**Figure 6:**
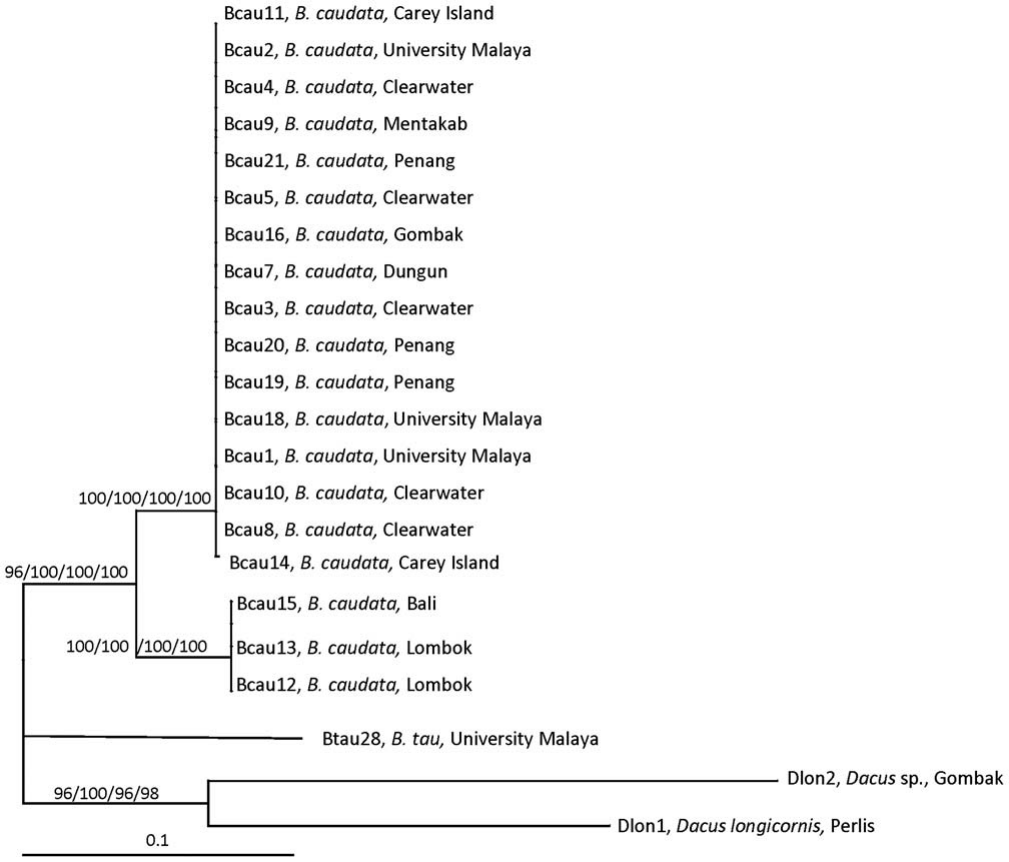
The 50% majority-rule consensus tree resulting from maximum likelihood analysis of combined COI and16S rDNA sequences (substitution rate parameters: TC = 0.7202736; TA = 0.07817598; TG = 0.005510263; CA = 0.07817598; CG = 0.005510263; AG = 0.112354). -Ln likelihood was 2726.135. The bootstrap values (ML/Bayesian Inference/MP/NJ) are shown at the branches. Bar indicates substitutions per site.

The ML tree for the combined COI and 16S rDNA sequences consisted of two main groups with *B. tau* as the basal group but with no bootstrap value. The second group consisted of two clades (Figure 6). Clade 1 consisted of *B. caudata* from various localities from Malaysia supported with full bootstrap values for all analyses. Clade 2 consisted of *B. caudata* from Bali and Lombok (Nusa Tenggara, Indonesia) and also supported with full bootstrap values for all analyses.

## Discussion

Among the component species of the subgenus *Zeugodacus* of the genus *Bactrocera* of tephritid fruit flies, distinct genetic lineages (cryptic species) have been found in *B. ascita* [9] and *B. tau* [12] based on COI sequences. The present finding of distinct genetic lineages in *B. caudata* based on COI and 16S sequences increases the number in the list.

The type locality of *B. caudata* is Java, Indonesia. However it had not been recorded to be present in Bali and Lombok [34] as well as Sulawesi [35]. The present specimens of *B. caudata* from Bali and Lombok are genetically clearly different from *B. caudata* of Malaysia and other parts of mainland Asia. Based on COI sequences, the uncorrected ‘p’ distance between *B. caudata* of Malaysia-Thailand-China and *B. caudata* of Bali-Lombok (Indonesia) was 5.65% (Table 1). The genetic distance based on 16S sequences ranged from 2.76 to 2.99% (Table 1). For the combined COI and 16S dataset, the genetic distance ranged from 4.45 to 4.46% (Table 2). These values for COI and 16S as well as the combined dataset were clearly different from the intraspecific values (‘p’ = 0–0.23). Furthermore, they are comparable to the genetic distance between related species of the subgenus *Zeugodacus*, e.g.‘p’ = 1.15% for 16S between *B. cucurbitae* and *B. tau*, and ‘p’ = 0.94% for COI sequences between *B. diaphora* and *B. scutellata* (Table 1) – these ‘p’ values are the lowest between two distinct species.

Based on the main morphological characters (black band across the face, three yellow postsutural vittae and the costal band slightly enlarged at the apex) the Bali and Lombok specimens concur with the description of *B. caudata*. There are no distinct differences in other gross morphological characters that have been used for taxonomic determination. As in the case of *B. ascita* sp. A and *B. ascita* sp. B as well as *B. tau* complex, a detailed study based on bigger samples and specimens from various localities in Indonesia as well as other parts of the distribution range is needed to delimit the occurrence of *B. caudata* and determine the extent of distinct genetic lineages (or cryptic species). In particular, attention should be given to the taxa found in Sumatra, Java and Flores [2], [34].

It is noteworthy that *B. caudata* from Indonesia is found in two adjacent islands, Bali and Lombok. Biogeographically, Bali is part of the Sundaland while Lombok is in Wallacea. However the close proximity of the two islands could easily facilitate the spread of the fruit fly from one island to the other through particularly movement of infested host plants. Studies are needed to determine the distribution of this species east of Bali and west of Lombok. Earlier studies based on COI [12] and COI and 16S sequences [11] indicated distinct separation of *B. caudata* from the group consisting of *B. cucurbitae* and *B. tau*. The present analysis concurs with these findings. Indeed *B. tau*, *B. cucurbitae*, *B. cucumis* (a member of subgenus *Austrodacus*) and *B. depressa* form a distinct clade from the other species.

In the present study which included species (e.g. *B. ascita*, *B. scutellata*, *B. ishigakiensis*, *B. depressa*) not treated together in earlier studies [9], [11], [36], [37], the phylogenetic analysis based on COI sequences indicated that *B. ascita* sp. B was the closest relative of *B. caudata* and was clearly separated from *B. ascita* sp. A which formed a clade with *B. scutellata*, *B. ishigakiensis* and *B. diaphora* (Figure 4). The analysis based on COI sequences (Figure 4) indicated that *B. ascita* sp. B was a sister group to *B. caudata* while *B. ascita* sp. A grouped with *B. scutellata*, *B. ishigakiensis* and *B. diaphora* (Figure 4). In an earlier study [37], without the inclusion of *B. ascita* and *B. caudata*, *B. scutellata* and *B. ishigakiensis* formed a clade with *Bactrocera* sp. (Japan).

In summary, this study has demonstrated distinct genetic lineages (or cryptic/sibling species) in *B. caudata*. Whether there exist more distinct genetic lineages (or cryptic species) in different parts of its distribution range needs to be studied. This study also confirms the usefulness of COI and 16S markers for species differentiation and phylogenetic determination.

## References

[1] Norrbom AL (2004). Updates to biosystematic database of world Diptera for Tephritidae through 1999.. Diptera Dissemination Disk (CD-ROM).

[2] Carroll LE, White IM, Freidberg A, Norrbom AL, Dallwitz MJ (2002). Pest fruit flies of the world.. http://delta-intkey.com.

[3] White IM, Elson-Harris MM (1992). Fruit flies of economic significance: their identification and bionomics.

[4] Drew RAI, Robinson AS, Hooper G (1989). The taxonomy & distribution of tropical and subtropical Dacinae (Diptera: Tephritidae).. Fruit flies, their biology, natural enemies and control. World Cro Pests 3(A).

[5] Drew RAI (1989). The tropical fruit flies (Diptera: Tephritidae: Dacinae) of the Australasian and Oceanian regions.. Memoirs of the Queensland Museum.

[6] Asian Fruit Fly IPM Project (2012). Field exercise guide on fruit flies integrated pest management.. http://ipm.ait.asia.

[7] Hardy DE (1973). The fruits flies (Tephritidae – Diptera) of Thailand and bordering countries.. Pacific Insects Monograph.

[8] Yong HS, Ho YW, Vidyadaran MK, Abdullah N, Jainudeen MR, Bahaman AR (1992). Host specificity and genetic variability in Malaysian fruit flies (Insecta: Diptera: Tephritidae).. Proceedings of the National IRPA (Intensification of Research in Priority Areas) Seminar (Agriculture Sector), Volume 1: Crops and Plants.

[9] Jamnongluk W, Baimai V, Kittayapong P (2003). Molecular evolution of tephritid fruit flies in the genus *Bactrocera* based on the cytochrome oxidase I gene.. Genetica.

[10] Smith PT, Kambhampati S, Armstrong KA (2003). Phylogenetic relationships among *Bactrocera* species (Diptera: Tephritidae) inferred from mitochondrial DNA sequences.. Molecular Phylogenetics and Evolution.

[11] Zhang B, Liu YH, Wu WX, Wang ZL (2010). Molecular phylogeny of *Bactrocera* species (Diptera: Tephritidae: Dacini) inferred from mitochondrial sequences of 16S rDNA and COI sequences.. Florida Entomologists.

[12] Jamnongkluk W, Baimai V, Kittayapong P (2003). Molecular phylogeny of tephritid fruit flies in the *Bactrocera tau* complex using the mitochondrial COI sequences.. Genome.

[13] Mun J, Bohonak AJ, Roderick GK (2003). Population structure of the pumpkin fruit fly *Bactrocera depressa* (Tephritidae) in Korea and Japan: Pliocene allopatry or recent invasion?. Molecular Ecology.

[14] Shi W, Kerdelhue C, Ye H (2005). Population genetics of the Oriental fruit fly, *Bactrocera dorsalis* (Diptera: Tephritidae), in Yunnan (China) based on mitochondrial DNA sequences.. Environmental Entomology.

[15] Hu J, Zhang JL, Nardi F, Zhang RJ (2008). Population genetic structure of the melon fly, *Bactrocera cucurbitae* (Diptera: Tephritidae), from China and Southeast Asia.. Genetica.

[16] Roderick GK (1996). Geographic structure of insect population: gene flow, phylogeography, and their uses.. Annual Review of Entomology.

[17] Simon C, Frati F, Beckenbach A, Crespi B, Liu H (1994). Evolution, weighting and phylogenetic utility of mitochondrial gene sequences and a compilation of conserved polymerase chain reaction primers.. Annals of Entomological Society America.

[18] Lunt DH, Zhang DX, Szymura JM, Hewitt GM (1996). The insect cytochrome oxidase I gene: evolutionary patterns and conserved primers for phylogenetic studies.. Insect Molecular Biology.

[19] Swofford DL (2002). PAUP*: Phylogenetic analysis using parsimony (*and other methods). Version 4.

[20] Clement M, Posada D, Crandall KA (2000). TCS: a computer program to estimate gene genealogies.. Molecular Ecology.

[21] Templeton AR, Crandall KA, Sing CF (1992). A cladistic analysis of phenotypic associations with haplotypes inferred from restriction endonuclease mapping and DNA sequence data. III. Cladogram estimation.. Genetics.

[22] Thompson JD, Gibson TJ, Plewniak F, Jeanmougin F, Higgins DG (1997). The ClustalX windows interface: flexible strategies for multiple sequence alignment aided by quality analysis tools.. Nucleic Acids Research.

[23] Huelsenbeck JP, Bull JJ, Cunningham CW (1996). Combining data in phylogenetic analysis.. TREE.

[24] Whelan S, Liò P, Goldman N (2001). Molecular phylogenetics: state-of-the-art methods for looking into the past.. Trends in Genetics.

[25] Mickevich MF, Farris JS (1981). The implications of congruence in Menidia.. Systematic Zoology.

[26] Farris JS, Källersjö M, Kluge AG, Bult C (1994). Testing significance of incongruence.. Cladistics.

[27] Farris JS, Källersjö M, Kluge AG, Bult C (1995). Constructing a significance test for incongruence.. Systematic Biology.

[28] Yoder AD, Irwin JA, Payseur BA (2001). Failure of the ILD to determine data combinability for slow loris phylogeny.. Systematic Biology.

[29] Jobb G, von Haeseler A, Strimmer K (2004). Treefinder: a powerful graphical analysis environment for molecular phylogenetics.. BMC Evolutionary Biology.

[30] Huelsenbeck JP, Ronquist F (2001). MrBayes: Bayesian Inference of Phylogenetic Trees.. Bioinformatics.

[31] Tanabe AS (2007). Kakusan: a computer program to automate the selection of a nucleotide substitution model and the configuration of a mixed model on multilocus data.. Molecular Ecology Notes.

[32] Akaike H, Petrov BN, Csaki F (1973). Information Theory and an Extension of the Maximum Likelihood Principle.. Second International Symposiumon Information Theory.

[33] Shono H (2000). Efficiency of the finite correction of Akaike's information criteria.. Fisheries Science.

[34] Hardy DE (1983). The fruit flies of the genus *Dacus* Fabricius of Java, Sumatra and Lombok, Indonesia (Diptera: Tephritidae).. Treubia.

[35] Hardy DE (1982). The Dacini of Sulawesi (Diptera: Tephritidae).. Treubia.

[36] Muraji M, Nakahara S (2001). Phylogenetic relationships among fruit flies, *Bactrocera* (Diptera: Tephritidae), based on mitochondrial DNA.. Insect Molecular Biology.

[37] Nakahara S, Muraji M (2008). Phylogenetic analyses of *Bactrocera* fruit flies (Diptera: Tephritidae) based on nucleotide sequences of the mitochondrial COI and COII genes.. Research Bulletin Plant Protection Japan.

